# Vitamin D Status Differs by Sex, Sport-Season, and Skin Pigmentation among Elite Collegiate Basketball Players

**DOI:** 10.3390/sports7110239

**Published:** 2019-11-18

**Authors:** Jennifer B. Fields, Daniel C. Payne, Sina Gallo, Deanna R. Busteed, Margaret T. Jones

**Affiliations:** 1 Frank Pettrone Center for Sports Performance, George Mason University, Fairfax, VA 22030, USA; 2 School of Kinesiology, George Mason University, Manassas, VA 20110, USA; 3 Nutrition and Food Studies, George Mason University, Fairfax, VA 22030, USA

**Keywords:** college athletes, 25(OH)D, indoor sports

## Abstract

Vitamin D plays a key role in bone health, musculoskeletal function, and sport performance. Collegiate athletes competing in indoor sports may be at greater risk of vitamin D deficiency due to limited outdoor time. Therefore, the purpose was to assess 25-hydroxyvitamin D (25(OH)D) concentrations among collegiate men and women basketball (MBB, WBB) athletes. National Collegiate Athletic Association Division I men (MBB, *n* = 11) and women (WBB, *n* = 9) were tested during the off-season (T1; July) and pre-season (T2; October). Measurements included serum 25(OH)D; skin pigmentation, bone mineral density, and daily sun exposure (self-reported). Paired t-tests determined changes in 25(OH)D by sport-season and sex. Pearson correlations examined relationships between outcome variables. MBB athletes (mean ± SD; 19.6 ± 1.3 years) showed a reduction in 25(OH)D (T1: 64.53 nmol·L^−1^ ± 11.96) (T2: 56.11 nmol·L^−1^ ± 7.90) (*p* = 0.001). WBB (20.1 ± 1.1 years) had no change in 25(OH)D (T1: 99.07 nmol·L^−1^ ± 49.94. T2: 97.56 nmol·L^−1^ ± 36.47, *p* = 0.848). A positive association between 25(OH)D and skin pigmentation was observed (r = 0.47, *p* = 0.038). 25(OH)D was inversely correlated with lean body mass (LBM), body mass (BM), and bone mineral density (BMD), while a positive association was seen between 25(OH)D and skin pigmentation. In summary, 25(OH)D insufficiency was prevalent amongst male collegiate basketball athletes, with 25(OH)D levels being lower in the pre-season (October) than the off-season (July). Furthermore, darker skin pigmentation significantly correlated with 25(OH)D, indicating that individuals with darker skin tones may be at a greater risk of insufficiency/deficiency. More research is needed to examine the relationships between 25(OH)D and bone health in athletes.

## 1. Introduction

Vitamin D is a lipid-soluble vitamin that is responsible for a large number of processes within the human body [1], among them immune function, cell growth, bone health, and neuromuscular function [2,3]. 25-hydroxyvitamin D (25(OH)D) is used clinically to determine vitamin D status [2]. Vitamin D can be obtained through certain food products such as margarine, cereals, fortified milk, and fatty fish [1]. However, unlike other vitamins, vitamin D is unique in that it is synthesized endogenously through skin exposure to ultra-violet B (UVB) rays [3]. Factors that may prevent the number of UVB photons penetrating the skin can have a direct impact upon the rate of vitamin D synthesis. This can include the season of year, time of day, darker skin pigmentation, living at more northern latitudes (>35°N), and spending large periods of time indoors [4,5]. 

Along with an increased risk of bone fractures and osteoporosis [3], deficient levels of 25(OH)D have been linked to atrophy of type II muscle fibers [4], slower returns to pre-operative strength levels following ACL reconstructive surgery [6], and inhibited musculoskeletal performance [7]. Lower injury rates have been reported in National Football League (NFL) athletes with sufficient (>75 nmol·L^−1^) 25(OH)D levels [8] and in vitamin D supplemented ballet dancers [9]. Furthermore, low 25(OH)D status has significantly correlated with increased frequency of illness in National Collegiate Athletic Association (NCAA) athletes (r = −0.40, *p* = 0.048) [10]. 

Although 25(OH)D deficiency is a growing health concern for members of the general population, with approximately 1 billion people believed to be affected worldwide [11], minimal information exists with regard to the vitamin D status of collegiate athletes [12]. Previous research conducted in elite level athletes indicated deficient (<50 nmol·L^−1^) or insufficient (50–75 nmol·L^−1^) levels of 25(OH)D in ~80% of National Basketball Association (NBA) combined athletes (*n* = 221, deficient: 40.1 ± 5.2 nmol·L^−1^; insufficient: 62.4 ± 8.4 nmol·L^−1^) [13]. The risk of vitamin D deficiency extends to college-aged athletes, specifically those who compete in sports that primarily take place indoors, where exposure to sunlight is limited. Halliday et al. [10] found that collegiate athletes (*n* = 12) participating in indoor sports had lower circulating 25(OH)D in the autumn months than athletes participating in outdoor sports. Therefore, indoor athletes may be at a higher risk for deficiency, adversely affecting their health and performance; however, limited research has examined collegiate indoor sport athletes.

Since basketball is predominantly an indoor sport and these athletes engage in minimal outdoor activities, it was hypothesized that a high prevalence of vitamin D deficiency and insufficiency would be observed. Hence, the purpose of the present study was to assess 25(OH)D during the pre-season and off-season in both male and female National Collegiate Athletic Association (NCAA) Division I (DI) basketball players. Secondary aims of the study were to examine the relationship between 25(OH)D, bone mineral density, sun exposure, and skin pigmentation. It was hypothesized that greater rates of vitamin D deficiency would be associated with lower bone mineral density, lean body mass, and darker skin pigmentation. 

## 2. Materials and Methods

### 2.1. Study Design and Participants

A total of 20 NCAA DI male (MBB) and female (WBB) basketball players (MBB, *n* = 11, age: 19.64 ± 1.29 years; WBB, *n* = 9, age: 20.11 ± 1.01 years) participated. The athletes were healthy, resistance-trained, and currently on comparable training programs. All were following sport-specific training regimens and were involved in regular sport training activities with specific neuromuscular demands. All participants completed a medical history form and had been medically cleared for intercollegiate athletic participation. In order to draw comparisons across two seasons of the training cycle (summer off-season vs. autumn pre-season), data collection took place at two separate time points. The first scheduled collection (T1; summer off-season) occurred in July, while the second time point (T2; autumn pre-season) took place in October. During the summer off-season and autumn pre-season periods, basketball athletes trained indoors as a team, and followed the NCAA mandated guidelines for training sessions and total training time permitted. Since the half-life of 25(OH)D ranges from 15 days to three weeks [14], the July and October time points will reflect vitamin D status from summer and autumn, respectively. The Institutional Review Board for Human Subjects approved (IRB approval: 978815-4) all procedures. The risks and benefits were explained and an institutionally approved consent form was signed prior to participation.

### 2.2. Measurements

Upon arrival to the laboratory, participants completed self-report questionnaires, which included questions on demographics, sport experience, recent travel within the previous three months, and sunscreen use. Ethnicity was self-reported.

#### 2.2.1. Skin Pigmentation

Skin pigmentation was measured on the facultative forehead using a portable computerized spectrophotometer (CM-600D, Konica Minolta, Ramsey, NJ, USA). Based on the Commission Internationale de l’Eclairage colorimetry system (color spaces: L*a*b*), the individual typological angle (ITA) (ITA° = (arc tangent (L* − 50)/b*)) 180/3.14159) was calculated [15]. Athletes were classified into five skin phototypes: dark (≤10°), olive (20–28°), medium (28–41°), fair (41–55°), and very fair (>55°) [15].

#### 2.2.2. Sun Exposure

Sun exposure was quantified using the sun exposure questionnaire (SEQ) [16]. Participants were asked to indicate their travel to warmer climates within the past three months, average time per week exposed to sunlight, and sunscreen usage. 

#### 2.2.3. Body Composition and Bone Health

Dual energy x-ray absorptiometry (DXA; Hologic, Horizon A model, Marlborough, MA, USA) was used to assess body fat (BF%), fat mass (FM), lean body mass (LBM), and whole-body bone mineral density (BMD). Participants were instructed to wear standardized clothing with no metal parts (i.e., drawstring pants and t-shirt). The participants were instructed to remain motionless and breathe normally while scanning was in process [17]. All DXA scans were overseen by an International Society for Clinical Densitometry Certified Bone Densitometry Technologist. Radiation (~3.4 mSV) did not exceed limits for x-ray exposure. Prior to testing, a quality assurance phantom (Hologic phantom serial #26436) was performed to ensure calibration of bone. Weekly calibration for the body composition measures was performed using a whole-body phantom (Hologic #1104). The coefficients of variation established in our laboratory were 1.07%, 1.17%, and 9.88% for LBM, BF%, and FM, respectively. The coefficient of variation for BMD was 1.03%.

#### 2.2.4. Hematology and Blood Analysis

A fasted venous blood sample was collected by a registered nurse for analysis of serum 25(OH)D. Participants were seated in an upright position, with the antecubital area sterilized using standard sterile phlebotomy procedures. Blood samples were collected into a 3 mL vacutainer (BD Biosciences, San Jose, CA, USA) following venipuncture of the median cubital vein and were allowed to coagulate in cooling beds for ~30 min. Samples were then centrifuged at 2500 revolutions·min^−1^ for 15 minutes (Eppendorf 5702R, Eppendorf North America, Hauppauge, NY, USA) before being stored in aliquots at −80 °C for further batch analysis. 25(OH)D status was determined using an enzyme-linked immunosorbent assay (Monobind, Lake Forest, CA, USA). Intra-assay coefficient of variation for 25(OH)D was 3.7%. The following cut-offs were used to determine 25(OH)D (nmol·L^−1^) health status: ≤50 = deficient; 50–75 = insufficient; ≥75 = sufficient [16].

### 2.3. Statistical Analysis

SPSS version 25.0 (IBM, Armonk, NY, USA) was used for data analysis. Descriptive statistics including means and standard deviations were calculated for all continuous variables. Separate paired samples t-tests and Cohen’s d effect sizes for MBB and WBB were used to determine changes in body composition and vitamin D status from July (summer off-season, T1) to October (autumn pre-season, T2). Pearson correlations were used to examine relationships between body composition measures, 25(OH)D, sun exposure, and skin pigmentation. Moderate correlations were defined as R-values of 0.41–0.70 and strong correlations defined as R-values of 0.71–0.99 [18]. 

## 3. Results

**Figure 1:**
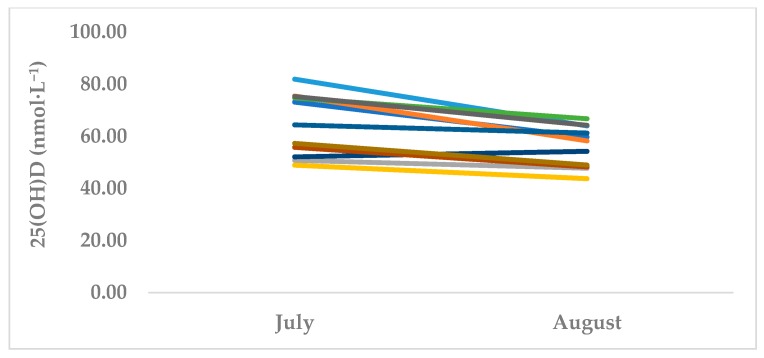
Serum 25(OH)D changes in MBB (*n* = 11). *Each line represents an individual MBB athlete. July is T1 (summer off-season) and October is T2 (autumn pre-season).

**Figure 2:**
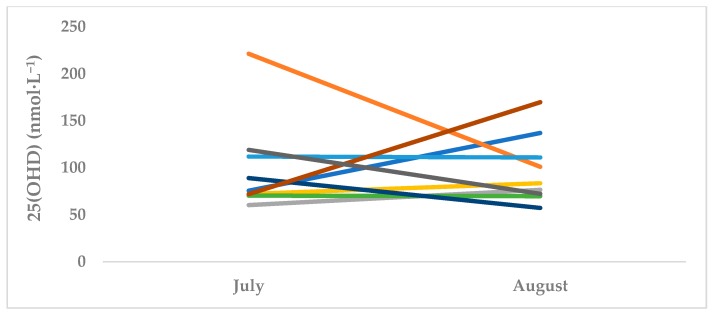
Serum 25(OH)D Changes in WBB (*n* = 9). *Each line represents an individual WBB athlete. July is T1 (summer off-season) and October is T2 (autumn pre-season).

MBB showed a significant reduction in 25(OH)D from T1 (summer off-season, July) (64.5 nmol·L^−1^ ± 11.9) to T2 (autumn pre-season, October) (56.1 nmol·L^−1^ ± 7.9, *p* = 0.001, d = 0.83) (Table 1). Conversely, WBB showed no change in 25(OH)D levels (99.1 nmol·L^−1^ ± 49.9 to 97.6 nmol·L^−1^ ± 36.5, *p* = 0.848, d = 0.04). (Table 2). At both T1 and T2, seven MBB athletes (63.6%) had 25(OH)D values in the insufficient range (Table 1). At T2, a total of four (36.3%) MBB athletes had deficient levels of serum 25(OH)D. While three (27.2%) subjects presented sufficient 25(OH)D levels at T1, no athletes had sufficient levels by T2. No WBB athletes had deficient 25(OH)D levels at either T1 or T2. At T1, four (44.4%) athletes were insufficient, while five (55.5%) participants had 25(OH)D levels in a sufficient range. At T2, three (33.3%) WBB subjects had insufficient levels while the remaining six (66.6%) athletes were sufficient. Figure 1 and Figure 2 represent individual MBB and WBB athlete responses from T1 to T2.

25(OH)D was moderately, inversely correlated with LBM (r = −0.569, *p* < 0.001), BM (r = −0.434, *p* = 0.005), and BMD (r = −0.411, *p* = 0.008), while a moderate positive association between 25(OH)D and skin pigmentation was also observed (r = 0.47, *p* = 0.038). No relationships were seen between sun exposure and 25(OH)D or BMD.

## 4. Discussion

Findings from the present study indicate a high prevalence of 25(OH)D insufficiency (<75 nmol·L^−1^) in MBB athletes during both the summer off-season (T1; July: *n* = 8, 74.7%) and autumn pre-season (T2; October: *n* = 11, 100%). In contrast, average serum 25(OH)D values for WBB were sufficient (>75 nmol·L^−1^) at both time points (T1 = 99.07, T2 = 97.56 nmol·L^−1^). Since measures were assessed at 38.8462°N, −77.27642°E during the summer and autumn, we hypothesized vitamin D status would be the highest. Further differing from our hypotheses, moderate inverse relationships were seen between 25(OH)D and LBM (r = −0.569, *p* < 0.001), BM (r = −0.434, *p* = 0.005), and BMD (r = −0.411, *p* = 0.008). However, 25(OH)D was positively associated with skin pigmentation (r = 0.470, *p* = 0.038).

In a separate study of NCAA athletes, Hildebrand et al. [11] found that 68% had adequate (>75 nmol·L^−1^) 25(OH)D levels. However, 46% of these athletes competed in an outdoor sport (e.g., soccer, baseball, softball, football), where sun exposure is greater. Our findings support previous research with MBB athletes (*n* = 279) [13] that reported approximately 80% had insufficient (<80 nmol·L^−1^) 25(OH)D levels. Barcal et al. [19] reported similar findings amongst male collegiate wrestlers, with 94% of their sample (*n* = 14) having insufficient 25(OH)D levels (<80 nmol·L^−1^) in both the winter and spring seasons. However, National Hockey League professional athletes (*n* = 105) did not show high levels of deficiency (13%, <80 nmol·L^−1^), although the assessment took place once at the end of summer and may not reflect 25(OH)D status at other time points in the year. Furthermore, their findings were obtained through medical records that did not provide detailed information with regard to sun exposure prior to blood draw. In contrast to the MBB athletes in the present study, 25(OH)D insufficiency was not seen amongst our cohort of WBB athletes. This observation is in contrast to previous reports of 25(OH)D in female athletes, where insufficient levels had been reported in 83% of gymnasts [20], 40% of endurance trained athletes [19], and 58% of elite track and field athletes [21]. While 25(OH)D status did not differ between sexes in past studies with endurance [22] or collegiate athletes [10,23], findings from the present study demonstrated that the majority of WBB players were able to maintain sufficient 25(OH)D status in both the summer off-season and autumn pre-season, while their male counterparts were insufficient at both time points. We speculate that the difference in 25(OH)D levels may have been attributed to the ethnicity of the MBB (i.e., 91% were African American) and WBB athletes (i.e., 56% white Anglo). Dark skin tone has been shown to be a significant predictor of abnormal 25(OH)D levels in NCAA athletes [24]. Although dietary intake of vitamin D was not analyzed, the difference observed between sexes might be attributed to the dietary intake of vitamin D. 

Similar to findings of the current study, seasonal variation in 25(OH)D has been reported with collegiate [10,25] and elite level athletes [26,27,28,29]. Previous research conducted in Japanese collegiate athletes found that those competing in indoor sports (basketball and volleyball; *n* = 14) had lower 25(OH)D levels in the winter (59.9 nmol·L^−1^) and spring (47.4 nmol·L^−1^) than in the summer (72.3 nmol·L^−1^) and autumn (79.8 nmol·L^−1^) [25]. Similar findings have been observed in collegiate athletes from the United States, with Halliday and colleagues [10] noting that 60.6% of NCAA athletes (18 men/23 women; 12 indoor/39 outdoor) had insufficient 25(OH)D levels (<80 nmol·L^−1^) in the winter compared to 9.8% and 16% in the autumn and spring, respectively. In a separate study with Dutch sub-elite athletes, who primarily competed in an indoor sport (*n* = 45, 86.5%), Backx et al. [29] reported a 36% decrease in 25(OH)D levels from the end of the summer season (September; 113 nmol·L^−1^) to the end of the winter season (March; 78 nmol·L^−1^). This fluctuation in 25(OH)D levels across seasons is thought to be caused by the sun’s zenith angle, which is more oblique during the winter months, and subsequently leads to a large amount of UVB photons being absorbed by the Earth’s ozone layer. As a result, minimal vitamin D_3_ is produced in the skin during the winter season [3]. As indicated by previous research and supported by findings of the current study with MBB athletes, this decrease in 25(OH)D during the later seasons of the year (autumn, winter) may be greater for athletes competing in an indoor sport. We speculated that the lack of seasonal variation seen in WBB may be due to a difference in lifestyle habits during the off-season period. WBB athletes may have been exposed to more quality sunlight and consumed a balanced diet rich in sources of dietary vitamin D. However, dietary intake of vitamin D was not assessed in the present study, making it difficult to draw definitive conclusions. 

In the present study, 25(OH)D status was negatively correlated with LBM, while no relationships were seen with FM and BF%. These results are in contrast with previous findings from both the general population and athletes [23,30,31,32]. The inverse relationship between 25(OH)D and FM seen in previous studies supports the fat sequestration hypothesis, where vitamin D is preferentially stored in adipose tissue instead of being transported into the bloodstream where it can perform metabolic functions [33]. However, Forney et al. [12] did not witness a relationship between 25(OH)D and BF% in physically active, college-aged students (*n* = 39: 20 men, 19 women). Furthermore, 25(OH)D status did not significantly correlate with BMI, BM, or FM in a separate cohort of NCAA collegiate athletes (*n* = 41: 18 men, 23 women) [10]. This lack of association may be due to reduced levels of body fat seen in physically active individuals compared with sedentary population groups [10]. Future studies using larger sample sizes of athlete populations are needed to examine the relationship between 25(OH)D and body composition.

In contrast to our initial hypothesis, we observed a negative association between 25(OH)D and whole-body BMD. While a positive relationship between 25(OH)D and BMD has been consistently reported in the general population [34], research with a large sample of athletes (*n* = 950) reported no association between 25(OH)D and BMD at the hip, neck, and spine [35]. Due to the role of 25(OH)D on calcium absorption and bone remodeling, deficient levels of 25(OH)D have been attributed to excessive bone turnover and an increased risk of osteoporosis and stress fractures [34,36,37]. However, the relationship between 25(OH)D and BMD may be sport-dependent. Athletes participating in sports that require greater bone loading through multi-directional impact (e.g., gymnastics, volleyball, basketball) have presented greater BMD than those participating in non-weight bearing activity (e.g., swimming) [38]. Frequent exposure to high impact, bone loading activities appears to influence the osteogenic response, thus increasing BMD in the whole body and at specific sites [39]. Therefore, past relationships between 25(OH)D and BMD seen in sedentary populations may not carry over to physically active individuals competing in high impact sports such as basketball. 

A significant, positive association between 25(OH)D and skin pigmentation was seen in the present study, indicating that darker skin tones may be associated with 25(OH)D insufficiency. Villacis et al. [24] observed a similar relationship amongst a cohort of NCAA DI athletes (*n* = 223), with abnormal 25(OH)D levels being 15.2 times greater in athletes with a dark skin tone. A separate study with elite track and field athletes from the United Kingdom [21] reported lower 25(OH)D levels in dark-skinned compared to fair-skinned athletes. The increased risk of 25(OH)D insufficiency in dark-skinned athletes is thought to be caused by greater amounts of melanin within the epidermal layer of the skin [40]. Melanin efficiency absorbs UVB photons, reducing the capacity for the skin to synthesize vitamin D_3_. Thus, it has been suggested that individuals with darker skin pigmentations require longer exposure to sunlight in order to synthesize the same amount of vitamin D_3_ as light-skinned individuals [3]. Interestingly, sun exposure did not correlate with 25(OH)D in the current study, and no association has been seen in populations living in countries with abundant sun exposure [41,42]. This lack of correlation between sun exposure and 25(OH)D may be due to a number of factors. Diligent application of sunscreen, residing at more northern latitudes (>35°N), and skin covering clothes can all limit the ability to synthesize vitamin D_3_ from sunlight [3,41,42], suggesting that sun exposure may not be indicative of 25(OH)D status. 

The relationship between vitamin D status (25(OH)D), BMD, body composition (LBM, FM, BF%), and skin pigmentation in a sample of NCAA DI male and female collegiate basketball athletes was examined in the present study. Insufficient (<75 nmol·L^−1^) 25(OH)D levels were seen in MBB athletes and levels were significantly reduced between the summer off-season and autumn pre-season. While a small sample size (*n* = 20) makes it difficult to draw steadfast conclusions between 25(OH)D and measures of body composition, this study was strengthened by the assessment of 25(OH)D at two separate time points within the training cycle. 

In summary, we found that 25(OH)D insufficiency was prevalent amongst male collegiate basketball athletes, with 25(OH)D levels being lower in the autumn pre-season (October) than the summer off-season (July). Further, darker skin pigmentation significantly correlated with 25(OH)D, indicating that individuals with darker skin tones may be at a greater risk of insufficiency/deficiency. Due to the importance of 25(OH)D upon health and sports performance, it is recommended that practitioners working with basketball athletes consider routine monitoring of 25(OH)D status. Doing so may guide nutrition professionals in their recommendations for vitamin D supplementation, as standards in optimal intake are lacking. Additional supplementation may aid in improving athletic performance and overall health. Based on the current results, it is suggested that the relationship between 25(OH)D and body composition measures be investigated further in high level athletes.

## Figures and Tables

**Table 1:** Serum 25(OH)D concentrations in MBB at T1 and T2 (*n* = 11).

Characteristics	T1 (Summer Off-season, July) Mean ± SD	T2 (Autumn Pre-season, October) Mean ± SD
25(OH)D (nmol·L^−1^)	64.5 ± 11.9	56.1 ± 7.9 *
<50 nmol·L^−1^	1 (11.1%)	4 (36.3%)
50–75 nmol·L^−1^	7 (63.6%)	7 (63.6%)
>75 nmol·L^−1^	3 (27.2%)	0 (0%)

* *P* < 0.05. 25(OH)D cut-off data reported as n (%).

**Table 2:** Serum 25(OH)D concentrations in WBB at T1 and T2 (*n* = 9).

Characteristics	T1 (Summer Off-season, July) Mean ± SD	T2 (Autumn Pre-season, October) Mean ± SD
25(OH)D (nmol·L^−1^)	99.1 ± 49.9	97.6 ± 36.5
<50 nmol·L^−1^	0 (0%)	0 (0%)
50–75 nmol·L^−1^	4 (44.4%)	3 (33.3%)
>75 nmol·L^−1^	5 (55.5%)	6 (66.6%)
